# Multifunctional Scatterometer System for Measuring Physical Oceanographic Parameters Using Range-Doppler FMCW Radar

**DOI:** 10.3390/s22082890

**Published:** 2022-04-09

**Authors:** Ji-Hwan Hwang, Duk-jin Kim, Ki-Mook Kang

**Affiliations:** 1 Research Institute of Basic Sciences, Seoul National University, Seoul 88026, Korea; hwang1651@snu.ac.kr; 2 School of Earth and Environmental Science, Seoul National University, Seoul 88026, Korea; 3 Water Resources Satellite Research Center, K-Water Research Institute, Daejeon 34045, Korea; mook0416@kwater.or.kr

**Keywords:** multifunctional scatterometer, FMCW radar, range-doppler process, ocean observation

## Abstract

A multifunctional scatterometer system and optimized radar signal processing for simultaneous observation of various physical oceanographic parameters are described in this paper. Existing observation methods with microwave remote sensing techniques generally use several separate systems such as scatterometer, altimeter, and Doppler radar for sea surface monitoring, which are inefficient in system operation and cross-analysis of each observation data. To improve this point, we integrated separate measurement functions into a single observation system by adding a measurement function of Doppler frequency to the existing system. So it enables to simultaneously measure the range and polarimetric responses of backscattering as well as movements of the sea surface. Here, the simultaneous measurement function of Doppler frequency was implemented by sampling an FMCW (frequency modulated continuous wave) radar signal as 2D raw data consisting of fast- and slow-time samples, i.e., the range and backscattering of radar target signals are analyzed from the fast-time samples while the Doppler frequency by the radar target’s movement extracts from the slow-time samples. Through the Fourier transformed-based range-Doppler signal process, distance (*R*), backscattering (*σ°*), and Doppler frequency (*f_D_*) are sequentially extracted from the 2D raw data, and a correlation to the physical oceanographic parameters is analyzed. Operability of the proposed system was examed through total 3 times of field campaigns from June 2017 to August 2020 and the observation data retrieved by the radar measurement data (*R*, *σ°*, *f_D_*) was also cross-analyzed with in-situ data: e.g., tide, significant wave height, and wind speed and direction. Differences in the comparative results as an observational accuracy are as follows. Tidal level (Root Mean Square Error 0.169 m (*R*)), significant wave height (RMSE 0.127 m *(R)*, 0.362 m (*σ*°)), wind speed (RMSE 1.880 m/s (*f_D_*), 2.094 m/s (*σ*°)) and direction (18.84° (*f_D_*)).

## 1. Introduction

Recently, global climate change has had a great impact on the marine environment, and the physical effects of climate change on the ocean cause sea level rise which will affect coastal areas, ocean currents, weather, and the seafloor environment. The correlation between ocean temperature rise and sea-level rise has been proven through continuous ocean observations since the last century, and observation data collected by various observation techniques were used as basic data for analysis of changes in the maritime environment. So, many researchers have traditionally observed physical oceanographic parameters (e.g., tide level, wave height, ocean current vector, wind vector, ocean temperature, salinity, and density, etc.) and have recently used various microwave remote sensing techniques for analysis of these parameters through sea surface monitoring, as well [1,2,3,4,5,6,7].

The radar-based ocean observation system is improving the efficiency and accuracy of observation by measuring the physical oceanographic parameters complimentarily using a stationary ocean platform-based system and a satellite or aircraft-based system. In particular, the ocean platform-based system not only has the advantage of continuously observing a specific area but also can perform a role as a test site for calibration of satellite or aircraft observation data [8,9,10]. However, because the existing radar-based systems operated in the ocean platform generally use separate observation systems such as scatterometers, altimeters, and Doppler radars to measure each physical oceanographic parameter, there are aspects of inefficiency in system operation and cross-analysis of data. In addition, because the ocean platform has many constraints of installation or operation for the separate observation systems, the integrated observation system improved operational efficiency is more needed. If it can simultaneously measure the radar measure parameters such as distance (*R*), backscattering (*σ°*), and Doppler frequency (*f_D_*), the existing separate systems will be replaced with a single system. The proposed multifunctional scatterometer system can be a good candidate to resolve this point because the simultaneous observation function can be implemented by just adding a measure function of Doppler frequency to the existing scatterometer system, which can measure the target’s distance and backscattering intensity. It can also provide the time-series observation data, which is not required to synchronize in cross-analysis among the measure parameters.

In this study, to continuously observe changes in the marine environments at the ocean platform, development of the scatterometer system to measure various oceanographic data simultaneously and research on ocean monitoring techniques were conducted. To effectively implement the system, the FMCW (frequency modulated continuous wave) signal-based radar transceiver was employed because of the advantage of sampling de-chirped signals using a relatively low sampling rate and the ease of implementing the receiver [11,12,13]. Furthermore, each radar parameter can be sequentially extracted through the Fourier transform-based range-Doppler process, which is theoretically derived from the FMCW signal model and validated using the simulation and practical data [14,15]. Especially, extracting the Doppler frequency from the raw data measured by the proposed system requires a faster sampling frequency than the existing system only for measuring distance or backscattering intensity, where the resolvable Doppler frequency span is dependent on the PRF (pulse repetition frequency) which is the repetition times of the existing sampling frequency. The received signals sampled by a faster sampling frequency are arranged as 2D raw data consisting of fast- and slow-time samples, then applying the Fourier transform-based range-Doppler process enables the analysis of distance, backscattering intensity as well as Doppler frequency. Then, to acquire accurate backscattering coefficients (*σ°*), calibration process such as STCT (single target calibration technique) and DMMCT (differential Mueller matrix calibration technique) was applied [16,17], and the calibration accuracy was verified with the scattering model of IEM (integral equation scattering model) [18,19]. Furthermore, in this process, the performance of the self-manufactured radar transceiver was verified through a comparative experiment using a network analyzer (Anritsu MS2028B). The radar measure parameters (*R*, *σ°*, *f_D_*) measured by the proposed system supporting the X-band frequency(9.4~9.9 GHz) with vertical polarization can be converted into various physical oceanographic parameters, which had verified through cross-validation of in-situ data such as tidal level, significant wave height, wind speed and direction. Total 3 times of the field campaigns had conducted at the Ieodo ocean research station, which is located about 150 km far from Jeju island of Korea, and whole processes including system operation and data analysis were validated [20].

## 2. FMCW Signal Model and Range-Doppler Processing

**Figure 1:**
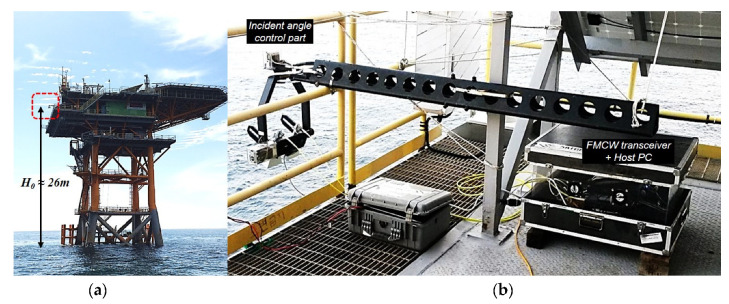
Overview of the FMCW radar-based multifunctional scatterometer system: (**a**) the Ieodo ocean research station and (**b**) the scatterometer system operating for ocean monitoring.

Acquiring the 2D raw data of the FMCW radar-based multifunctional scatterometer system as shown in Figure 1 is theoretically implementable using a repetitive measure function with a specific slow-time sampling frequency (e.g., PRF 100 Hz), it is added to the existing signal processing of FMCW radar that analyzes the target’s delay time (*τ*) from the beat frequency (*f_b_*) and can be referred to SAR (synthetic aperture radar) signal model [14,15]. In other words, this function that can repetitively measure the range profiles with a specific interval enables to detection of the target movement within a specific range because slow-time sampling frequency is proportional to Doppler frequency. Therefore, the 2D raw data consisting of fast- and slow-time samples are sequentially transformed into the range-Doppler domain, then we can analyze the radar measure parameters such as distance, backscattering intensity, and Doppler frequency. The signal transformation process of the 2D raw data is theoretically derived using the FMCW signal model, and it is verified by simulation using a point target. Furthermore, through this process, the system requirements of the proposed FMCW radar transceiver are validated.

### 2.1. FMCW Signal Model

Tx- and Rx-signals of the FMCW signal-based scatterometer system can be theoretically derived using a linear FMCW signal model, here, the 2D signal model includes the slow-time (*η*) domain for analysis of Doppler frequency (*f_D_*). The ideally linear FMCW signal has a frequency modulation characteristic of 1st order function with respect to a variable of *t* (refer to fast-time bins), and it is described as a phase function integrated by a variable of *t*, as follows. Equation (1) is a normalized phase function of Tx signal, *s_t_(t,η)*, where *f*_0_ is a center frequency, *K_r_* is a chirp rate, *ϕ* is an initial value of phase, and *t* and *η* are the fast- and slow-time bins, respectively. Equation (2) is the received signal, *s_r_(t,η)*, from the target located at *τ = 2R*/*c* (*R* target distance, *c* light speed) and includes the signal amplitude function, *a(t,η)*, which is propotional to the polarimetric target response. Equation (3) is the intermediate frequency (IF) Rx signal down-converted by the reference Tx signal, it includes the beat frequency components to analyze a range profile of the interest target.

(1) $$s_{t}\left(t,\eta \right)=\exp\left\{-j\left(2\pi f_{0}t+\pi K_{r}t^{2}+\phi \right)\right\}$$

(2) $$s_{r}\left(t,\eta \right)=a\left(t,\eta \right)\cdot \exp\left\{-j\left(2\pi f_{0}\left(t-\tau \right)+\pi K_{r}{\left(t-\tau \right)}^{2}+\phi \right)\right\}$$

(3) $$s\left(t,\eta \right)=s_{r}\left(t,\eta \right)\cdot s_{t}^{*}\left(t,\eta \right)=a\left(t,\eta \right)\cdot \exp\left\{j\left(2\pi f_{0}\tau +2\pi K_{r}t\tau -\pi K_{r}\tau^{2}\right)\right\}$$

Equations (1)–(3) are the Tx/Rx and intermediated signals of the LFM signal-based CW radar system, and the difference from the existing model is that a variable of the slow-time (*η*) is added for repetitive range-profile data acquisition. It can be compared to the SAR signal model [14], but, in this study, it is used for the purpose of detecting the movement of a target on a fixed platform.

Equation (4) is the first process of the Fourier transformation to analyze target distance (*R =* *τ·c*/*2*) from the intermediated Rx signal of Equation (3) including the beat frequency component (*f_b_ =* *K_r_·τ*). The variable of fast-time (*t*) in 2D fast- and slow-time sampled data is transformed into the variable of frequency (*f_r_*) for the range domain, the first transformed signal *S(f_r_,η)* is consisting of an amplitude function and a phase function. The amplitude function described as a sinc function is proportional to the polarimetric target response and the position is shifted as much as the beat frequency (*f_b_*), and the phase function expressed as an exponential function has the phase component proportional to target time-delay (*τ*) (refer to the final line of Equation (4)).

(4) $$\begin{array}{l} S\left(f_{r},\eta \right)=\int_{-T_{r}/2}^{T_{r}/2}s\left(t,\eta \right)\cdot \exp\left\{-j2\pi f_{r}t\right\}\;dt\\ =\frac{\exp\left\{j\left(\pi f_{r}-\pi K_{r}\tau \right)T_{r}+j2\pi f_{0}\tau -j\pi K_{r}\tau^{2}\right\}}{j\left(2\pi f_{r}-2\pi K_{r}\tau \right)}-\frac{\exp\left\{-j\left(\pi f_{r}-\pi K_{r}\tau \right)T_{r}+j2\pi f_{0}\tau -j\pi K_{r}\tau^{2}\right\}}{j\left(2\pi f_{r}-2\pi K_{r}\tau \right)}\\ =T_{r}\cdot \left[\frac{\exp\left\{j\pi \left(f_{r}-K_{r}\tau \right)T_{r}\right\}}{2j\pi \left(f_{r}-K_{r}\tau \right)T_{r}}-\frac{\exp\left\{-j\pi \left(f_{r}-K_{r}\tau \right)T_{r}\right\}}{2j\pi \left(f_{r}-K_{r}\tau \right)T_{r}}\right]\cdot \exp\left\{j2\pi f_{0}\tau -j\pi K_{r}\tau^{2}\right\}\\ =T_{r}\cdot \sin c\left\{\left(f_{r}-K_{r}\tau \right)T_{r}\right\}\cdot \exp\left\{j2\pi f_{0}\tau -j\pi K_{r}\tau^{2}\right\}\\ ≅T_{r}\cdot \sin c\left\{\left(f_{r}-f_{b}\right)T_{r}\right\}\cdot \exp\left\{j2\pi f_{0}\tau \right\} \end{array}$$

Particularly, the phase function of the transformed signal *S(f_r_,η)* includes a second order of phase term (*−jπK_r_·τ*^2^), so-called the RVP (residual video phase), it may be a source of the skewness effect of the sampled data proportional to *τ^2^* [21]. Therefore, it needs to pay attention to verify the signal model. Fortunately, because the observational distance of the proposed scatterometer system in this study is relatively short within less than 50 m, the skewness effect is very small and ignorable. Thus, the transformed signal can be approximated to the sinc function shifted as much as the beat frequency and the phase function of the propagated wave proportional to target time-delay (*τ*). In addition, frequency bins of the transformed signal can be rescaled by the beat frequency to range ratio (*f_b_*/*R = 2K_r_*/*c*), the frequency bins are replaced with the range bins.

Using the same manner, the second Fourier transformation can be applied to the variable of slow-time (*η*), where the phase function is keeping the same phase term for the propagated wave and a new sinc function is added in the direction of slow-time frequency (*f_η_*), as shown in Equation (5). Here, the transformed signal *S(f_r_, f_η_)* is for a stationary target; i.e., target time-delay (*τ*) is constant. So, the variable of frequency (*f_η_*) transformed from the slow-time (*η*) is used to analyze the Doppler frequency (*f_D_*), the Doppler shift becomes zero for this case.

(5) $$\begin{array}{ll} S\left(f_{r},f_{\eta }\right) & =T_{r}\cdot \sin c\left\{\left(f_{r}-f_{b}\right)T_{r}\right\}\cdot \int_{-T_{\eta }/2}^{T_{\eta }/2}\left[\exp\left\{j2\pi f_{0}\tau \right\}\cdot \exp\left\{-j2\pi f_{\eta }\eta \right\}\right]\;d\eta \\ & =T_{r}T_{\eta }\cdot \sin c\left\{\left(f_{r}-f_{b}\right)T_{r}\right\}\cdot \sin c\left\{f_{\eta }T_{\eta }\right\}\cdot \exp\left\{j2\pi f_{0}\tau \right\} \end{array}$$

On the other hand, for a moving target, the target time-delay (*τ*) is a function of the slow-time (*η*), the amount of change in target delay time (*δτ(η)*) during the measurement period (*T_η_*) can be described as Equation (6). Furthermore, a result of the Fourier transform reflecting this is as shown in Equation (7).

(6) $$\tau \left(\eta \right)=\tau_{0}+\delta \tau \left(\eta \right)=\frac{2R_{0}+2v_{t}\eta }{c}$$

(7) $$\begin{array}{ll} S\left(f_{r},f_{\eta }\right) & =T_{r}\cdot \sin c\left\{\left(f_{r}-f_{b}\right)T_{r}\right\}\cdot \int_{-T_{\eta }/2}^{T_{\eta }/2}\left[\exp\left\{j2\pi f_{0}\left(\tau_{0}+\delta \tau \left(\eta \right)\right)\right\}\right]\cdot \exp\left\{-j2\pi f_{\eta }\eta \right\}\;d\eta \\ & =T_{r}T_{\eta }\cdot \sin c\left\{\left(f_{r}-f_{b}\right)T_{r}\right\}\cdot \sin c\left\{\left(f_{\eta }-f_{D}\right)T_{\eta }\right\}\cdot \exp\left\{j2\pi f_{0}\tau \right\} \end{array}$$

The Doppler frequency (*f_D_ =* *f_0_·2v_t_*/*c*) proportional to a velocity (*v_t_*) of the moving target is transformed to a sinc function reflected Doppler shift of the same amount (*f_D_*) by the Fourier transform. Therefore, through the range-Doppler signal process as Equations (4)–(7), we can simultaneously analyze the center distance (*R*_0_) and Doppler frequency (*f_D_*) of the moving target, and the target velocity can be calculated back.

### 2.2. Range-Doppler Signal Processing

Next, the range-Doppler signal process applied to the practical scatterometer system is explained in this section. As mentioned before, the range-Doppler signal processing is basically conducted on the 2D sampled raw data by the Fourier transform, and the target’s distance (*R_0_*), Doppler frequency (*f_D_*), and polarimetric response such as backscattering coefficient (*σ°*) are extracted from them. 

**Figure 2:**
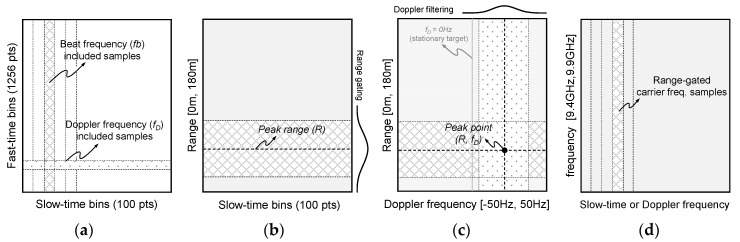
FFT based 2D raw data processing and data formats: these figures show (**a**) 2D raw data (fast-/slow-time samples), (**b**) 100 range-gated range profiles after 1st FFT applied to fast-time samples, (**c**) range-Doppler map after 2nd FFT applied to slow-time samples, and (**d**) 100 frequency responses of radar target after 3rd FFT applied to (**b**) or (**c**), respectively.

Figure 2 shows a procedure of the FFT-based signal process on the 2D raw data. Figure 2a is the 2D raw data consisting of the fast- and slow-time, same size (1252 × 100 samples) of raw data are applied for all incident angle conditions of the scatterometer system. Figure 2b shows a result of the first Fourier transform applied to the fast-time bins, where the range-gating is applied to increase the accuracy of signal analysis in the range of interests. The range-gating technique widely used in the field of radar signal processing is used to remove the unwanted signal on the propagation path, and it is also valid for removing the harmonic signals of the beat frequency [11,12]. Figure 2c is the transformed data to the range-Doppler domain by the second Fourier transform, so-called range-Doppler map, and the target’s distance and Doppler frequency are intuitively detected by peak searching. When the peak searching is conducted, the Doppler filtering can be also applied to clearly distinguish a Doppler spectrum of the moving target [11]. Figure 2d is the inverse transformed data from the range domain to the carrier frequency domain, the backscattering coefficients are calibrated in this domain. Therefore, radar parameters such as the target’s distance, Doppler frequency, and backscattering coefficient are precisely analyzed through the FFT-based data processing, and it is equally used for analysis of oceanographic parameters.

The above-mentioned signal process was validated through a simulation reflecting the system setups, as shown in Table 1. This FMCW radar has a center frequency of 9.65 GHz, a bandwidth of 500 MHz, and a chirp rate of 500 × 10^9^ Hz/s. To sample the 2D raw data, the fast- and slow-time sampling frequencies of about 1.2 MHz and 100 Hz are applied, respectively. Furthermore, it assumes that a test target moves with a speed of 1 m/s and it is observed at the incident angle of 20°.

**Figure 3:**
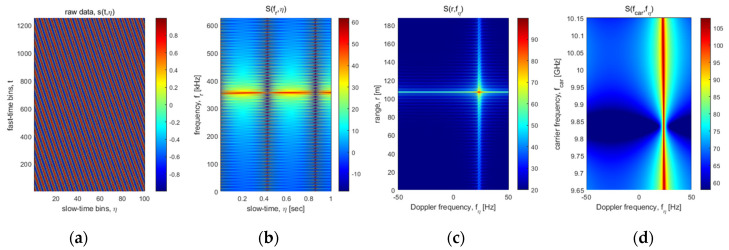
Simulation results for the range-Doppler signal process using a moving target with 1 m/s of velocity: these images show (**a**) fast-/slow-time sampled raw data, (**b**) 1st transformed frequency (*fr*) vs. slow-time (*η*) data, (**c**) 2nd transformed data, so-called range-doppler map, and (**d**) 3rd transformed carrier frequency (*fcar*) vs. Doppler frequency (*f_D_*) data, respectively.

Figure 3 shows simulation results sequentially transformed by the FFT-based signal process. Figure 3a is the 2D raw data generated by the FMCW signal model as Equation (3), Figure 3b is a beat frequency data applied the first FFT. In addition, Figure 3c is the range-Doppler map that target’s distance and velocity are intuitively detectable, characteristics of the moving target were analyzed to a distance of about 106 m and a velocity of about 1 m/s (e.g., *v_t_* *=* *c*/*2·f_D_*/*f_0_*/sin*θ_i_; f_D_* ≈ 23 Hz, *θ_i_ =* 20°), where it is the same values of simulation setup. Finally, Figure 3d is the transformed data to the carrier frequency domain for calibration of the scatterometer system and measurement data. The calibration process will be explained in the next section.

System requirements for design an FMCW transceiver of the proposed scatterometer system are referred to the specifications of Table 1. The maximum detection range (*R_max_*) was designed to be about 200 m, here, it depends on the fast-time sampling frequency (*f_ADC_*) and the chirp rate (*K_r_*) of FMCW signal [14,15]. In other words, the detectable maximum beat frequency is limited by half of the sampling frequency (*f_ADC_*). Thus, the DAQ board having enough sampling frequency (more than *f_ADC_ ×* *f_PRF_*) for 2D data acquisition is necessary, and the maximum range can be optimized by adjusting design parameters such as the chirp rate or the modulation period (*T_r_*) to the FMCW transceiver.

## 3. Self-Manufactured FMCW Scatterometer System

The proposed scatterometer system is roughly configured with an FMCW radar transceiver, a host-PC, and a mainframe of boom structure. The FMCW radar transceiver was adopted in consideration of manufacturing convenience and cost-effectiveness, and, for automatic observation and data management, all peripherals of the scatterometer system are controlled by a host PC. The details of the self-manufactured scatterometer system are as follows.

### 3.1. Self-Manufactured FMCW Radar Transciever

**Figure 4:**
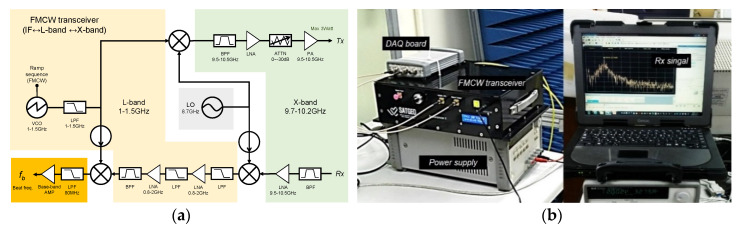
Self-manufactured FMCW radar system: (**a**) is a block diagram of the FMCW radar transceiver front-end circuit, and (**b**) shows an appearance of lab-test in progress.

Figure 4 shows a block diagram of the FMCW radar transceiver front-end circuit and an appearance of a lab test in progress. Figure 4a is the FMCW radar transceiver that 2-stage frequency up/down-converting circuit is added to a general homodyne receiver circuit; i.e., an additional local oscillator of 8.7 GHz enables to convert L-band (1~1.5 GHz) to X-band (9.4~9.9 GHz) and vice versa. In addition, by adding this 2-stage converting circuit, it is possible to effectively remove the noise of Tx/Rx modulated signals [11,12,13]. For LFM signal generation, the signal generator was composed of a VCO (voltage control oscillator) and a ramp sequence generator so that it is easy to generate and change a sawtooth waveform with a specific chirp rate. In Particular, the ramp sequence generator to control an output of VCO was designed in a memory-map method using a microprocessor (Microchips, PIC16F877A), which is widely used for education, and it generates the pre-distorted sawtooth waveform that has a vertical resolution of 12 bits (4096 steps). This transceiver transmits a pre-distorted LFM signal with a maximum power of 3 W and a chirp rate of 498 × 10^9^ Hz/s. Figure 4b shows The FMCW radar transceiver composed of a ready-made DAQ board (Keysight technologies, U2702A) and a host PC. Here, the fast- and slow-time sampling rate (e.g., *f_ADC_* ≈ 1.2 MHz, *f_PRF_* ≈ 100 Hz) was optimized in consideration of the data throughput between the DAQ board and the host PC.

### 3.2. Configuration of Scatterometer System

**Figure 5:**
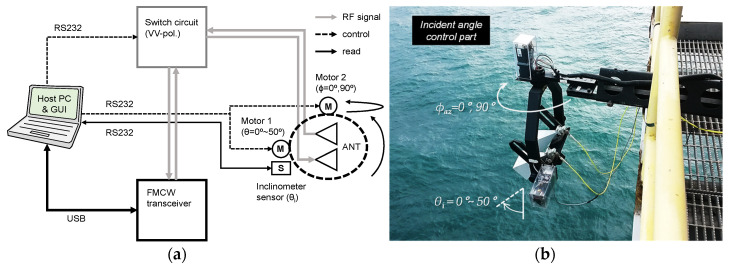
Configuration of the FMCW radar-based multi-functional scatterometer system: (**a**) is a block-diagram of the scatterometer system and (**b**) shows an appearance in working.

Figure 5 shows a configuration of the FMCW radar-based multifunctional scatterometer system. This self-manufactured scatterometer system has integrated all peripherals to a host PC and GUI (graphic user interface) for implementation of an automatic observation function. Particularly, In Figure 5a, the host PC can automatically control an incident angle within 0° to 50° and an azimuth angle within 0° to 90°, and it also automatically manages the raw data measured at each incident angle setup. To precisely adjust the incident angle, a digital inclinometer sensor (MicroStrain co., 3DM sensor) and 2 step-motors were applied, and the motion control algorithm that includes the functions to minimize or avoid the system errors due to high humidity marine environment was also self-programmed. Furthermore, all subfunctions such as the incident angle control, the raw data management, and the polarization switching are controlled by the optimized automatic measure sequence. In addition, Figure 5b shows the incident angle control part of the scatterometer system, which is movable in the directions of elevation or azimuth.

Table 2 shows the specification of the self-manufactured scatterometer system as follows. It transmits the linear FMCW signal, which has the center frequency of 9.65 GHz and the chirp rate of 498 × 10^9^ Hz/s, and collects the 2D raw data consisting of the fast- and slow-time samples. In addition, the scatterometer system having these performances and automatic measure function was equally applied to the field campaign for sea surface monitoring at the Ieodo ocean research station.

## 4. Operation of the Multifunctional Scatterometer System

Field campaigns using the self-manufactured scatterometer system had been performed at the Ieodo ocean research station for several years (June 2017, October 2018, August 2020). The scatterometer system was installed on the east side of the ocean platform, which is positioned at a height of about 26 m from the sea surface, and it was automatically operated to collect the observation data for about 1 week per field campaign. All measurement data were automatically collected by the optimized measure sequence, and also, it was precisely calibrated to improve the reliability of observation data. In this section, data acquisition and signal processing are described using the data measured in June 2017.

### 4.1. Automatic Measure Sequence

**Figure 6:**
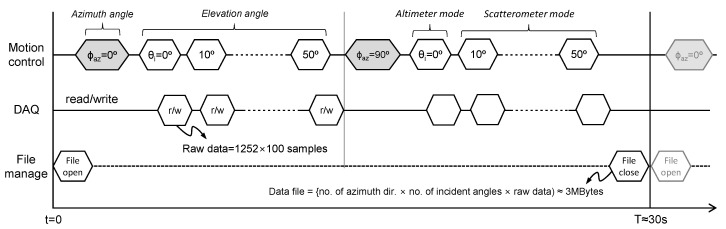
Automatic measure sequence of the multifunctional scatterometer system for sea surface monitoring; it includes 2-directional altimeter and scatterometer modes.

The raw data is automatically measured by the optimized measure sequence, as shown in Figure 6. This scatterometer system is operated under various incident angle conditions; i.e., 6 steps of the incident angle within 0° to 50°, 2 steps of the azimuth angle within 0° to 90° are moved sequentially. The raw data measured under these 12 conditions are automatically saved as a single data file of about 3 Mbytes. In addition, a single period of this measure sequence including the processing- or waiting-time takes about 30 s.

An operational concept of this scatterometer system includes 2 kinds of operating modes such as altimeter and scatterometer modes. It added the altimeter mode, which is not included in the existing system [22], to measure height or backscatter changes of the sea surface at the nadir angle (or the incident angle of 0°). In addition, the scatterometer mode measures the backscatters under the oblique incident condition as the old system. 2-directional observation conditions (e.g., azimuth look-angle of 0° or 90°; east or south) enable an analysis of different basis vector components on the analysis process of Doppler frequency, which serves the additional ocean observation information such as wind vector or current vector.

### 4.2. Calibration for Scatterometer System and Data

All measurement data of the scatterometer system should be precisely calibrated for analysis of the backscattering characteristics of the target. The radar calibration process is generally distinguished to the absolute calibration on the radar transceiver and the relative calibration on the antenna radiation pattern, and, using these calibration processes, the internal- or external distortion characteristic of the scatterometer system is compensated [16,17].

First, the absolute calibration process based on the STCT (single target calibration technique) begins with the analysis of the Rx signal reflecting the measurement distance (*r*^2^) for round trip manner and the distortion (*rt*) on the Tx/Rx signal path, as shown in Equation (8a) [16]. Since the multifunctional scatterometer system in this study is operated as a single polarization such as a vertical polarization (VV-pol.), the matrix formula for full-polarization is simplified to a single formula such as Equation (8a). The absolute calibration process is for compensating the distortion (*rt*) on the Tx/Rx signal path, so the Rx signal model of Equation (8a), which includes the phase and attenuation components proportional to the measurement distance with the distortion (*rt*), is used for the first calibration process. Equation (8b,c) are described for two kinds of measurement signals; i.e., the first Rx signal (*v_r,sph_*) of Equation (8b) is used for searching the internal distortion (*rt*) existing on the Tx/Rx signal path of the scatterometer system, where a conducting sphere is generally used as the reference target for calibration, and the second Rx signal (*v_r,test_*) of Equation (8c) is used for calibrating an RCS (radar cross-section) of the test target. From the two signals of Equations (8b,c), next equations such as Equation (8d,e) are derived, respectively. The unknown distortion (*rt*) is solved using the first measurement signal of Equation (8b), then, it is substituted into the unknown distortion value (*rt*) of the second measurement signal of Equation (8c). Here, unfortunately, the measured signal (*v_r,sph_* or *v_r,test_*) is not an s-parameter (*s_vv_ = v_r_*/*v_t_*) of the scattering matrix which is a ratio of the Rx signal over the Tx signal, because the Tx and Rx path of the self-manufacture FMCW radar transceiver is fully separated and the Rx signal only recorded. However, because the calibrated value should be also s-parameter, a more accurate calibration process in consideration of a change of the Tx signal (*v_t_*) should be added. Equation (8d) is the calibrated s-parameter of the test target in the case of a time-invariant Tx signal, whereas Equation (8e) is for the case of a time-varying Tx signal. It means that the calibrated result can be affected as much as the amount of change in the Tx signal (*v_t,sph_/v_t,test_*), and it should be compensated once again for accurate calibration. If this concept is applied to our system, as shown in Equation (8e), an optimized calibration process can be performed. So, as an alternative to estimate the amount of change in the Tx signal using a single DAQ board only, we propose an analysis technique using the range profile of the Rx signal.

(8a) $$s_{vv,meas}=\left(\frac{v_{r}}{v_{t}}\right)=\frac{e^{{}^{-j2kr}}}{r^{2}}\left(rt\right)\cdot s_{vv}$$

(8b) $$\begin{array}{ll} \to \;v_{r,sph}=\frac{e^{{}^{-j2kr_{0}}}}{r_{0}^{2}}\left(rt\right)\cdot s_{vv,\;sphere\;theory}\cdot v_{t,sph} & ;\;\mathrm{first}\;\mathrm{measurement}\;\mathrm{for}\;\mathrm{CAL}\;\mathrm{target} \end{array}$$

(8c) $$\begin{array}{ll} \to \;v_{r,test}=\frac{e^{{}^{-j2kr}}}{r^{2}}\left(rt\right)\cdot s_{vv,test}\cdot v_{t,test} & ;\;\mathrm{second}\;\mathrm{measurement}\;\mathrm{for}\;\mathrm{test}\;\mathrm{target} \end{array}$$

(8d) $$\to \;\mathrm{if}\;\left(v_{t,test}=v_{t,sph}\right),s_{vv,test}=\left\{\frac{r^{2}}{r_{0}^{2}}e^{{}^{-j2k\left(r_{0}-r\right)}}\right\}\cdot \left\{\frac{v_{r,test}}{v_{r,sph}}\right\}\cdot s_{vv,sphere\;theory}\;$$

(8e) $$\to \;\mathrm{if}\;\left(v_{t,test}\neq v_{t,sph}\right),s_{vv,test}=\left\{\frac{r^{2}}{r_{0}^{2}}e^{{}^{-j2k\left(r_{0}-r\right)}}\right\}\cdot \left\{\frac{v_{r,test}}{v_{r,sph}}\right\}\cdot \left\{\frac{v_{t,sph}}{v_{t,test}}\right\}\cdot s_{vv,sphere\;theory}$$

**Figure 7:**
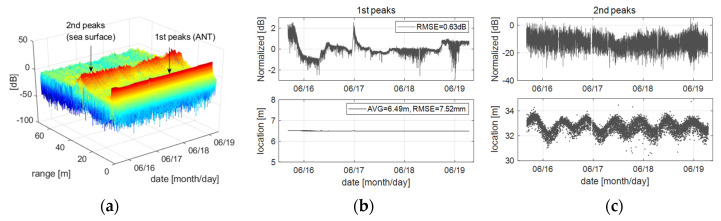
Analyzing a property of the time-varying peak signals extracted from the range profiles of altimeter mode: (**a**) is the time-series data consisting of about 8000 range profiles, and (**b**,**c**) is the first and the second peaks extracted from the figure (**a**), respectively.

Figure 7 is analyzing a time-series change of the range profiles of the Rx signals. Figure 7a shows the observational data converted into the range profile. All range profiles converted from measured Rx signal basically have two kinds of peak responses; the first peak is a returned signal from the antenna due to a discontinuity between conductor to air, and the second peak is a backscattered signal from the target such as sea surface. Here, the amount of change of the Tx signals can be estimated from the first peak. Since the scatterometer system including the antenna is physically invariant during operation, the change in the first peaks is due to the time-varying Tx signals. Thus, by analyzing the change in the first peaks, we can estimate the amount of change of the Tx signals without an additional DAQ board, as shown in Figure 7b. It shows the variance in the Tx signal normalized by a mean value of the first peaks. Additionally, through the change of locations of the first peaks, the stability of the modulated signal can be estimated indirectly. The FMCW radar transceiver has a phase delay proportional to the delay line of about 6.49 m including internal- and external delay terms due to the RF front-end circuit and antenna cables, and also the delay has the RMSE of 7.52 mm. It is inverted into the beat frequency by the beat frequency to range ratio (e.g., *f_b_*/*R ≈* 3.3 KHz/m), frequency error of the modulated signals is about 24 Hz over the beat frequency span of 625 KHz. In addition, Figure 7c shows the intensity and location of the second peaks that backscattered from the sea surface. The intensity implies the uncalibrated backscattering property of the sea surface, the locations give information on the change of wave heights. To acquire the calibrated backscattering coefficient (*σ*°) of the sea surface from the second peak signals, the relative (or radiation pattern) calibration process should be added. Since the oblique incident wave and the antenna beam pattern make a relative distortion within the footprint projected onto the sea surface, it is required to correct them. The relative calibration process is basically referred to as the differential Mueller matrix calibration technique and, it is summarized as Equation (9a) [17].

The Rx signal of DMMCT in Equation (9a) is modeled as a summation of the responses of differential scatterers, and it is added the relative distortion matrix (*D*) that occurred on the wave propagation path between the antenna and the differential scatterers to Equation (8a). In Equation (9a), the variable *a* is the distortion due to the Tx/Rx channel imbalance of radar transceiver such as the *rt* of Equation (8a), *D_vv_* is the distortion of the vertically polarized signal, where Equation (9a) is also simplified to as a single element of matrix formula. Equation (9b) is the calibration result of the Rx signal (*s_vv,meas_*) backscattered from the differential scatterers of the sea surface, and Equation (9c) is a formula to convert the calibrated s-parameter in Equation (9b) to the backscattering coefficient (*σ*°*_vv,sea_*), respectively. Here, calibration of the self-manufactured scatterometer system in consideration of the antenna radiation pattern should be performed using the reference data, which is precisely measured in an anechoic chamber because it is dependent on the calibration accuracy of the DMMCT.

(9a) $$s_{vv,meas}=\left\{\frac{v_{r}}{v_{t}}\right\}=\sum_{i}\sum_{j}\left\{a\cdot D_{vv}^{2}\left(x_{i},y_{j}\right)\cdot {s{}^{\circ}}_{vv,sea}\left(x_{i},y_{j}\right)\right\}$$

(9b) $$\to {s{}^{\circ}}_{vv,sea}=\frac{1}{N}\frac{1}{dxdy}\cdot \sum_{i}\sum_{j}\left[D_{vv}^{-2}\left(x_{i},y_{j}\right)\left\{\frac{r_{ij}^{2}}{r_{0}^{2}}e^{{}^{-j2k\left(r_{0}-r_{ij}\right)}}\right\}\cdot \left\{\frac{v_{r,sea}}{v_{r,sph}}\right\}\cdot s_{vv,sphere\;theory}\right]$$

(9c) $$\to {\sigma {}^{\circ}}_{vv,sea}=4\pi {\left|{s{}^{\circ}}_{vv,sea}\right|}^{2}$$

**Figure 8:**
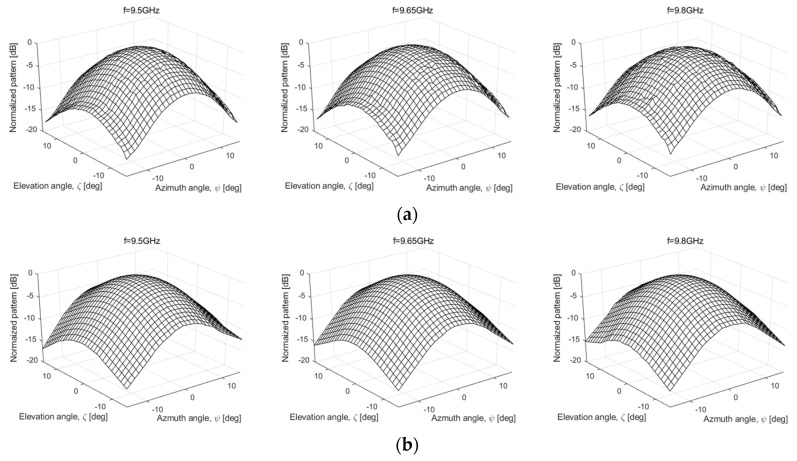
Comparison results of the reference radiation patterns of Tx/Rx antennas measured for the relative (or pattern) calibration: these data were measured by (**a**) the self-manufactured scatterometer system and (**b**) a network analyzer (Anritsu MS2028B).

Figure 8 shows a comparison result of the reference radiation patterns measured by our scatterometer system and network analyzer (Anritsu MS2028B). The measurement result was extracted from the data converted to the carrier frequency domain, and measured in the elevation- and azimuth-angle directions within ±15° using the automatic target scanning function of the scatterometer system [23]. It is the examples measured at three frequency (e.g., *f* = 9.5 GHz, 9.65 GHz, 9.8 GHz) within the operating frequencies, and the scatterometer data in Figure 8a is very consistent with them in Figure 8b.

**Figure 9:**
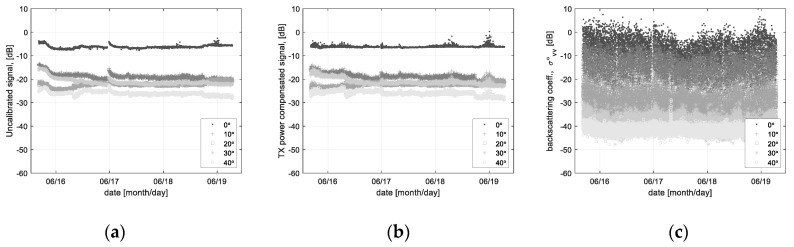
Backscattering coefficients (*σ*°*_vv_*) calibrated by the modified calibration technique: (**a**) raw data before calibration, (**b**) Tx power variation compensated data, (**c**) backscattering coefficients after calibration.

Figure 9 shows the examples for the scatterometer system calibration and the backscattering coefficient calibration of the sea surface. Figure 9a is the uncalibrated data including the Tx power change, Figure 9b is the compensated data by the proposed range profile analysis method (refer to Equation (8e) and Figure 7). Furthermore, Figure 9c is the calibrated backscattering coefficients, where it is the results of the entire calibration process such as the absolute- and relative calibration.

## 5. Analysis of Measurement Data: (Signal Process and Data Correlation)

The radar measure parameters (*R*, *σ*°, *f_D_*) are transformed from the raw data collected by the proposed multifunctional scatterometer system and inversed into the physical oceanographic parameters corresponding to the attribute of each radar parameter. For example, wave height changes can be directly analyzed using distance (*R*) information, while Doppler frequency (*f_D_*) can be inversely transformed into observational information about sea surface motion. In addition, the calibrated backscattering coefficient (*σ°*) can be used as in-situ data for research on existing theoretical scattering models or cross-analysis of satellite data.

In this section, we analyze the correlation of the ocean observation items (e.g., tidal level, significant wave height, wind vector, etc.) that can be inversely transformed from the three types of radar parameters. In addition, cross-validation with the theoretical scattering model according to sea state is performed using the calibrated backscattering coefficient (*σ*°).

### 5.1. Distance (R) vs. Wave Height

To extract the distance (*R*) information from the 2D raw data, Fourier transformation is applied as shown in Equation (4) to the measurement data of the altimeter operation mode of nadir angle (refer to Figure 7). In the altimeter mode, because microwaves are incident perpendicularly to the sea surface, and changes in ocean wave height can be directly analyzed from the radar measure parameter.

**Figure 10:**
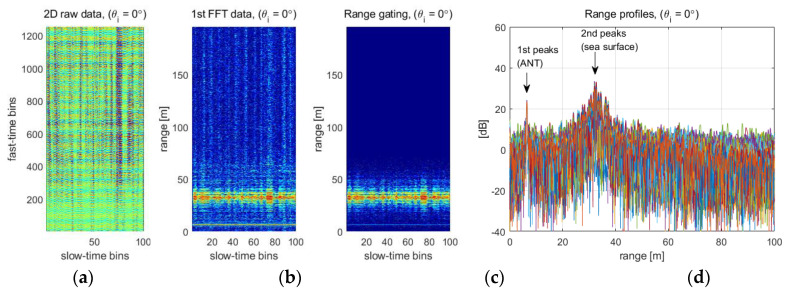
Extracting the distance (*R*) between antenna and sea surface from the altimeter mode data: (**a**) fast-/slow-time sampled raw data, (**b**) a transformed data to range/slow-time domain, (**c**) a range-gated data, and (**d**) 100 range profiles.

Figure 10 is an example showing the signal conversion and distance information extraction process using the actually measured raw data. Figure 10a is a 2D raw data, and Figure 10b,c are the results of applying Fourier transformation and range-gating for analyzing the target distance, respectively. In particular, Figure 10d shows 100 range profiles accumulated for target signal analysis in the region of interest, and two peak signals returned from the antenna and sea surface can be identified. Therefore, the change of ocean wave heights can be directly converted from the change in the relative distance between the two peaks.

The wave height that is one of the ocean observation items can be easily calculated by subtracting the average platform height (*H*_0_) from the relative distance (*R*) between the two peaks. So, an additional inversion algorithm is unnecessary for data conversion. Furthermore, to accurately analyze the wave heights, the 100 range profiles can be statistically used to improve stability or accuracy.

**Figure 11:**
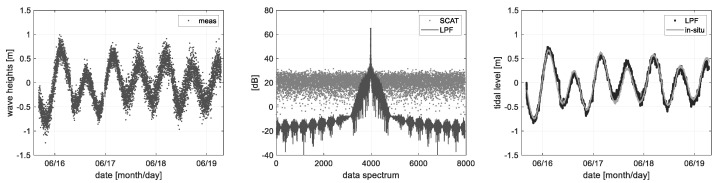
Tidal level change analyzed from a low-frequency component of the measured ocean wave.

**Figure 12:**
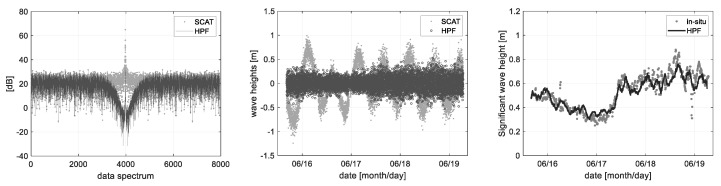
Significant wave heights (Hsig = 4 × σ) are processed statistically from the wave height data, where the low-frequency tidal components were removed.

The wave heights that were calculated from the radar distance information can be distinguished to the ocean observation items such as tidal level and significant wave height by the spectrum analysis method, as shown in Figure 11 and Figure 12. The measured wave height includes the tidal component of low frequency so that a pure wave height component such as the significant wave height can be filtered by the digital filter process applying an optimum cutoff frequency. To do this, the tidal- and significant wave height components can be precisely distinguished by applying the low- and high pass filter for each component.

Figure 11 is the result of the tidal level change analyzed from the wave height data measured by the altimeter mode. The tidal level change which is the low-frequency component in the wave height change can be analyzed by the low pass filtering. In the above example, the cutoff frequency of 1% of spectrum bandwidth was applied, the analysis result has an RMSE (root mean square error) of 0.0983 m in comparison to the in-situ data of the Ieodo ocean research station.

Whereas Figure 12 shows the high pass filter process to acquire a pure wave height component removing the tidal component. The significant wave height is statistically analyzed from the actual wave height change; i.e., the significant wave height (*H_sig_*) is 4 times of a standard deviation (*4σ*) of data within a specific time span. It has an RMS error of 0.0962 m in comparison to the in-situ data of the Ieodo ocean research station. In summary, the wave height changes can be analyzed from the radar parameter of distance (*R*), and it is possible to precisely distinguish the tide and significant wave height by the digital filter process.

### 5.2. Doppler Frequency (f_D_) vs. Wind Speed and Direction

**Figure 13:**
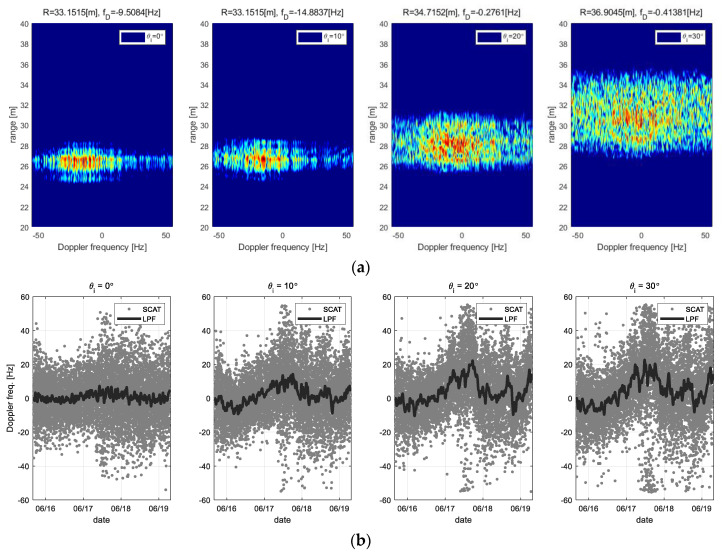
Extracting the Doppler frequency from Range-Doppler maps with the various incidence angle setups (e.g., *θi* = 0° ~ 30°): (**a**) is an example that the range-doppler signal process was applied, and (**b**) is a time-series data for analyzing the Doppler frequency shift.

Doppler frequency component is extracted from the 2D raw data measured under various incident angles, then the sea surface movements are analyzed from them. The range-Doppler map, which is processed from the 2D raw data by the Fourier transform, is as shown in Figure 13. Figure 13a is examples of the range-Doppler map to measure the Doppler frequency, where the optimized range-gating function for each incident angle was applied. In addition, Figure 13b shows the responses corresponding to change in sea surface movements as examples of the Doppler frequency analysis, where the Doppler frequency is the frequency component of a maximum peak signal searched from in the range-Doppler map.

In above Figure 13, the changes of the Doppler frequency corresponding to sea surface movements shows a similar trend of each response. This indicates that the Doppler frequency is responding to sea surface movement. Particularly, in the case of the oblique incident angle setup excepted for the nadir angle, the measured Doppler frequency data includes the vertical- and horizontal components of the sea surface movements, and the Doppler frequency due to the horizontal movements becomes dominant as increasing the incident angle. Thus, amplitude of the trends is also increasing. However, in the case of the nadir angle (*θ_i_* = 0°), the meaningful statistical analysis is difficult about the horizontal movement because the Doppler frequency indicates only the vertical component of sea surface movements.

**Figure 14:**
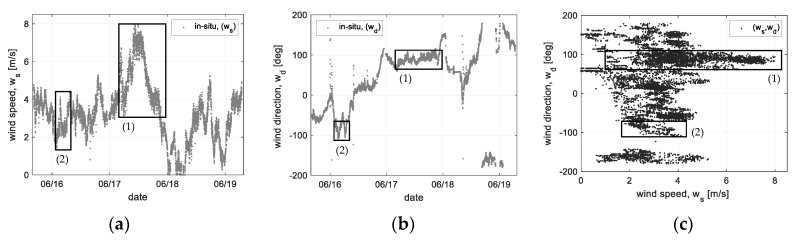
In-situ data of sea surface wind measured at 10 m height of Ieodo ocean research station: (**a**) a time-series wind speed, (**b**) a time-series wind direction, and (**c**) a reassigned data as wind speed vs. wind direction.

In addition, a correlation to ocean wind can be analyzed using the Doppler frequency data. For analysis of the correlation between the measured Doppler frequency and ocean wind, the in-situ data measured for the same period is applied. Here, the in-situ data was measured by the AWS (automatic weather system) sensor which was installed at the 10 m height from the sea surface. Figure 14 shows the in-situ data of ocean wind including wind speed (*W_s_*) and direction (*W_d_*) and the reassigned data (*W_s_,W_d_*) to analyze the correlation, respectively. Using this reassigned in-situ data, two data regions for the correlation analysis can be distinguished, as shown in Figure 14c. The region (1) and (2) are, respectively, separated as the wind direction of East and West. East wind of 1~7 m/s blew for an observation period of the region (1) and West wind of 1.5~4 m/s blew for another observation period of the region (2). Using these two types of observation data, the correlation between ocean wind (speed/direction) and Doppler frequency was analyzed as follows.

The two types of in-situ data, which the East (75° < Wd < 105°) and West (−75° < Wd < −105°) winds are selected in Figure 14, are compared with the Doppler frequency measured by the scatterometer system. Here the azimuth look angle kept the East side (*ϕ_az_* = 0°). Under this observation condition, the change of the Doppler frequency can be analyzed clearly; i.e., it may be an optimum condition to analyze the response characteristic of the Doppler frequency due to forward- and backward winds. The correlations to wind speed were analyzed using the selected data (1) and (2) in Figure 14. The linear regression method is applied to analyze the correlation between wind speed and Doppler frequency. At this time, the regression coefficient was analyzed from the data (*LPF*) that low-pass filtering to the reassigned data (*W_s_, f_D_*), and the fitting line was optimized by applying the least square method.

**Figure 15:**
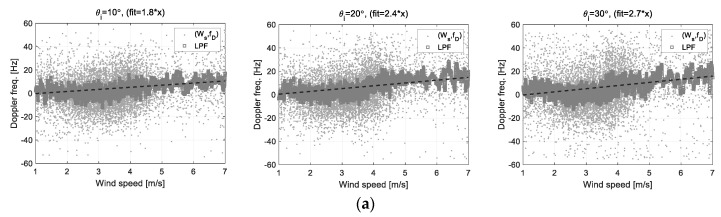

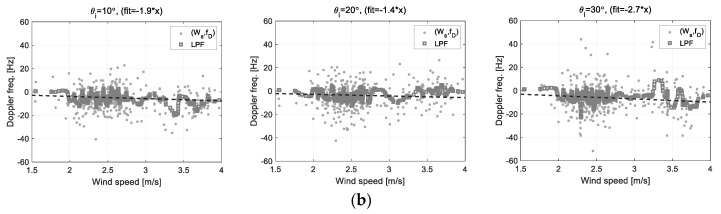
Correlations of the Doppler frequency data and the wind speed data analyzed by the linear regression method: these figures are the cases that when sea surface wind blew from (**a**) the east side or (**b**) the west side, the scatterometer looked at the Eastside (*ϕ_az_* = 0°).

Figure 15a shows the response characteristics of the Doppler frequency measured when the East wind blew in front of the scatterometer system, and Figure 15b shows the case when the West wind blew from the rear of the scatterometer system. These analysis data show that the absolute values of the regression coefficients generally increase as the incident angle increases in both data sets. Each regression coefficient is increasing within 1.8~2.7 or decreasing within −1.9~−2.7. However, in the case of Figure 15b, the number of samples during the observation period is small, so it may have limitations in the correlation analysis with relatively low reliability compared to Figure 15a. Despite this, it is confirmed that the Doppler frequency has a meaningful correlation to wind speed and the Doppler frequency shift is also highly correlated to wind direction. Therefore, based on these characteristics, it may be possible to estimate the ocean wind vectors using the observation data of Doppler frequency, and the results will be described in the next section.

### 5.3. Backscattering Coefficient (σ°) vs. Sea State

The measured data of Rx signal backscattered from the sea surface are calibrated to the backscattering coefficient (*σ*°) by the above-mentioned calibration process, and the polarimetric response corresponding to sea state is analyzed from this calibrated data. The sea state is generally classified to the WMO sea state code of 0~9, where it describes the general condition of the free surface on a large body of water due to wind and swells at a certain location and moment [24]. To analyze the sea state, the ocean wave spectrum model such as the Pierson-Moskowitz spectrum and the JONSWAP spectrum can be also referred [25,26]. However, estimating the sea state directly by the backscattering coefficient data is not easy because the microwave responds better to the wind or ocean wave as a source of the change of sea state. So, estimation of the sea state would rather analyze the correlation to the wind speed or the significant wave height. Therefore, to estimate the change of sea state, we can simultaneously analyze the correlation to the wind speed and the significant wave height, in addition, the polarimetric responses of the sea surface with a specific sea condition can be cross-validated using the IEM (integral equation scattering model), where is a well-known theoretical scattering model [18,19].

**Figure 16:**
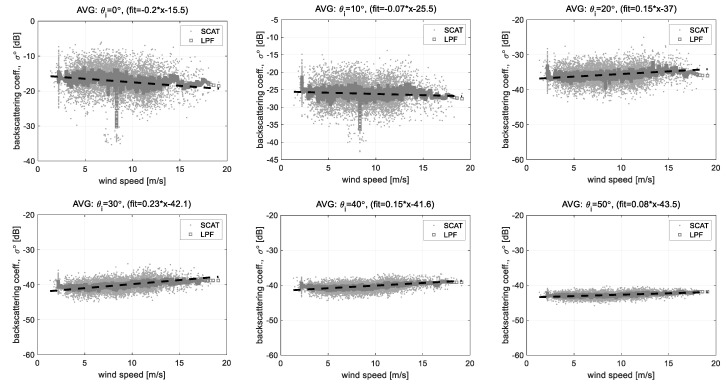
Correlations of the backscattering coefficient data and the wind speed data analyzed by the linear regression method: these figures are averaging the data measured at the East and South side (*ϕ_az_* = 0°, 90°).

Figure 16 is an example of the analysis result on the correlation between the measured backscattering coefficient within the incident angles of 0°~50° and the wind speed, and the scatterometer data measured on August 2008 was used. The wind speed measured for the observation period widely changed within about 3~17 m/s, and the measured backscattering coefficient data has a relatively uniform distribution within the wind speed of 5~15 m/s. Analysis results based on this effective range show that the regression coefficient roughly increases as the incident angle increases. This analysis result agreed well with a change of the polarimetric responses for each incident angle which is well known by theoretical scattering models [26]. In the case of the normal incident angle, the backscattering coefficient decreases as the wind speed increases. Whereas, in the case of the oblique incident angle within 20°~50°, the backscattering coefficient and wind speed are proportionally increasing together. The analysis result shows that the backscattering coefficient data measured under the incident angles of 20° and 30° are highly correlated with the wind speed and the normal incident wave data, so-called altimeter mode data, is also highly correlated but a negative sign. In addition, in the case of the incident angles of 40° and 50°, the regression coefficients rather decrease as the incident angle increases. This is related to a microwave property that the backscattering from the sea surface exponentially decreases as the incident angle increases because the forward scattering on the sea surface is dominant in a range of the incident angle of more than 30°, and also confirms that the backscattered Rx signal is measured at a near lower limit of the dynamic measurable range of the scatterometer system. In the future, the observation angle for ocean monitoring can be optimized within the incident angle of less than 30° to improve the operation efficiency of the multifunctional scatterometer system.

**Figure 17:**
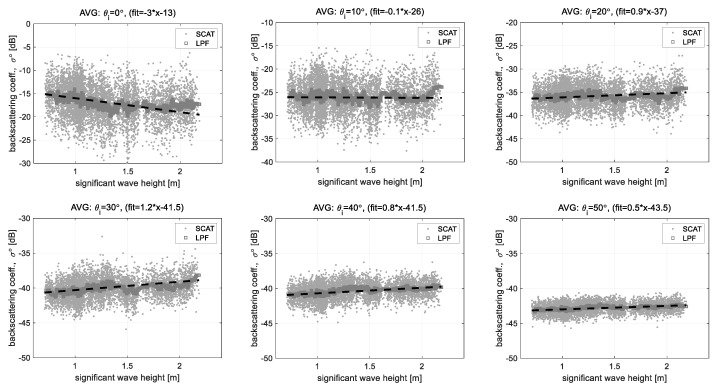
Correlations of the backscattering coefficient data and the significant wave height data analyzed by the linear regression method: these figures show a relatively low correlation with significant wave height because X-band microwave responds to the capillary wave of ocean waves well.

Next, Figure 17 shows an example of the analysis result on the correlation between the backscattering coefficients and the significant wave heights, using the same way of Figure 16. The significant wave heights measured for the same observation period changed within about 0.4~2.3 m, the measured backscattering coefficients have a relatively uniform distribution within less than 1.5 m of significant wave height because the high ocean wave was observed for a relatively short term. The analysis results show that the correlation to the significant wave height is roughly low compared to the case of wind speed. Particularly, in the case of the normal incident angle, the backscattering coefficients roughly decrease as the significant wave heights increase, however, a difference between the fitting line (dot line --) and the low pass filtered measurement data (LPF) is clear at outside of the effective range, more than the significant wave height of 1.5 m. The cases of the incident angle of 20° and 30° have also similar responses. This is related to the spectra property of ocean wave growth that the high frequency wave components such as the capillary wave due to ocean wind are accumulated and the accumulated energy of ocean wave gradually moves to a lower frequency component. In this process, it may be interpreted as a result that the operating frequency (*f*_0_ = 9.65 GHz, lam 0 ≈ 3 cm) of the scatterometer system responds better to a short wavelength ocean wave such as the capillary wave, and also stably responds to a relatively low wave height of less than 1.5 m. In addition, the measured signals under each incident angle are less contaminated due to decreasing the change of a local incident angle of scatterer as the wave height decreases. Therefore, analysis of the correlation between the backscattering coefficients and the significant wave heights using the X-band microwave is valid for the sea states of a relatively smooth or slightly rough surface, and further studies on data acquisition and analysis techniques for high wave heights are needed.

**Figure 18:**
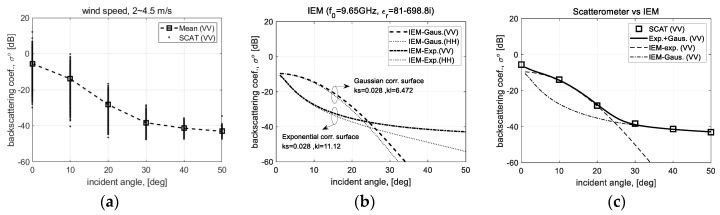
Comparing the polarimetric responses for each incident angle to validate the calibrated backscattering coefficient data to a theoretical scattering model of IEM-integral equation scattering model: (**a**) measured data, (**b**) scattering model data, and (**c**) comparative data.

The same backscattering coefficient data used as above can be cross-validated again with the theoretical scattering model. IEM (integral equation scattering model) was adopted for cross-validation, where the backscattering coefficient data measured for the observation period when the wave height is relatively low, e.g., less than code 3 of WMO sea state, was used [24]. Figure 18 shows a comparison result on the polarimetric responses for each incident angle between the backscattering coefficients and the calculated IEM data. Among the measurement data collected on June 2017, the measurement data only satisfying a specific observation condition, which is a wind speed of 2~4.5 m/s and significant wave height of less than 1 m, was selected. Figure 18a is the backscattering coefficient data for each incident angle calibrated from the 2D raw data of our scatterometer system, and Figure 18b is the simulation result using the IEM scattering model. A composite surface consisting of 2 types of randomly rough surfaces such as a Gaussian- and exponential correlated surface was generated, where each randomly rough surface is depicted by statistical characteristics such as RMS height (*ks*) and correlation length (*kl*). Furthermore, for simulation on the composite surface, the Gaussian surface of (*ks* = 0.028, *kl* = 6.472) and the exponential surface of (*ks* = 0.028, *kl* = 11.12) were generated, and the center frequency (*f*_0_) of 9.65 GHz and the relative permittivity (*ε_r_*) of 81-j698.8 were applied, respectively. Figure 18c is a comparison result of the measurement data and the simulation data as a polarimetric response for each incident angle. The simulation data for the slightly rough sea surface is matched well with the measured backscattering coefficient data.

## 6. Observational Results

In this section, we present examples of analysis results on tidal level, significant wave height, wind speed and direction from the radar measure parameters such as distance (*R*), Doppler frequency (*f_D_*), and backscattering coefficient (*σ*°). The various observation items can be simply converted using the linear correlation function analyzed previously, where the purpose of this study is for showing the observation capability of the scatterometer system rather than analyzing an observation accuracy. The scatterometer data, respectively, measured on June 2017, October 2018, August 2020, and the in-situ data measured for the same period were applied for cross-validation.

### 6.1. Tidal Level and Significant Wave Height

**Figure 19:**
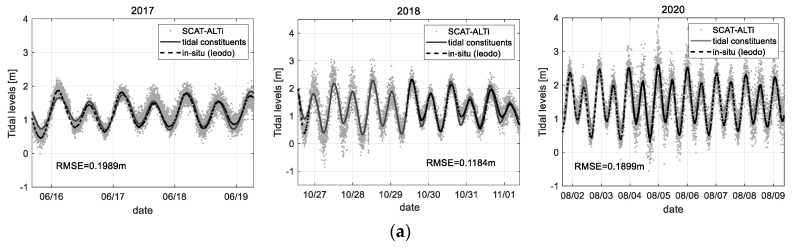

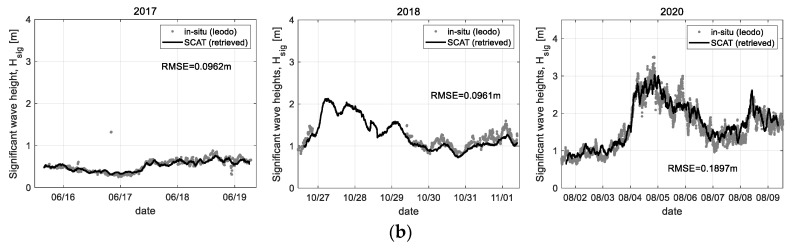
Observational results of (**a**) tidal level and (**b**) significant wave height retrieved by distance (*R*) data of the scatterometer system, where 2017, 2018, 2020 data were processed.

Figure 19 shows an analysis result on changes of tidal level and significant wave height for three times observation periods, in particular, where it used the distance (*R*) data sets analyzed from the 2D raw data measured by the altimeter mode. Figure 19a is the observation data for tidal level change monitoring. It is comparing the fitting curve approximated by 10 tidal constituents to the wave heights (‘SCAT-ALTi’) measured by the altimeter mode of the scatterometer system, and the Ieodo ocean research station’s own observation information (‘in-situ (Ieodo)’) was added for cross-validation. To analyze the main tidal constituents in the waters near the Ieodo station, the 10 tidal constituents as follows were applied to search the optimum values of amplitude (*H_n_*) and phase lag (*K_n_*). Using a tidal model of Equation (10) and the least square method, a total 4 of main tidal constituents were analyzed, where *ω_n_* is an angular speed of *n*-th partial tide and *A*_0_ is a mean sea level.

(10) $$H_{t}=A_{0}+\sum_{n}H_{\left(n\right)}\cos\left(\omega_{\left(n\right)}t-K_{\left(n\right)}\right)$$

Table 3 shows the analysis result using the 2018 data, where the 4 main tidal constituents (e.g., M2, S2, N2, K1) are easily distinguishable.

In addition, Figure 19b is the observation results for significant wave height, where these were statistically processed using 1 h of a time window. The significant wave height is equal to 4 times the standard deviation of wave height change, subtracted the tidal components, within the time window. Here, in particular, 2018 data has some faults which the Ieodo ocean research station’s data (‘in-situ’) could not measure the significant wave heights. Whereas, during this period of about two days, the significant wave height of more than 1.5 m could be measured by the scatterometer system. This will be a good example to show the advantages of the ocean observation system using the microwave remote sensing technique. In addition, all data confirm that it can effectively observe the ocean wave height change, where the accuracy is about RMSE 0.127 m on average.

### 6.2. Wind Speed and Direction

**Figure 20:**
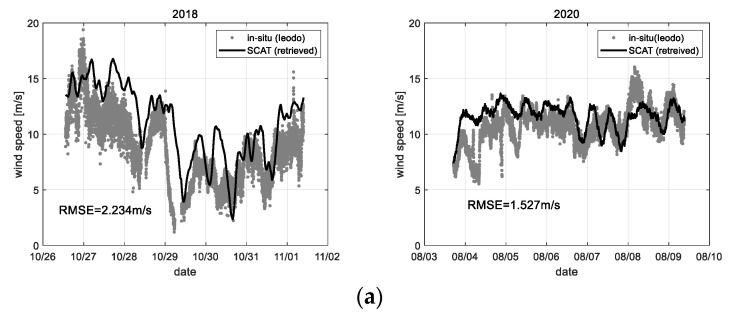

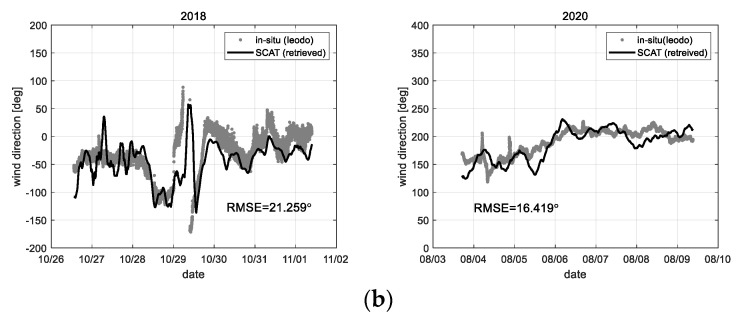
Observational results of (**a**) wind speed and (**b**) direction retrieved by Doppler frequency (*f_D_*) data of the scatterometer system, where 2018, 2020 data only were processed.

The wind vectors can be retrieved from the 2-axis Doppler frequency data measured on the east and south side, as shown in Figure 20. Where the data sets only for 2018 and 2020 were processed because the 2-axis measure function was added in 2018.

The Doppler frequency (*f_D_*) data collected through the range-Doppler map process can be inverted to the ocean wind speed and direction using the linear correlation function previously analyzed (refer to Section 5.2). To retrieve the wind speed and direction, the 2 types of Doppler frequency data sets give basis vectors of each azimuth direction, so the wind speed (*W_s_*) and the wind direction (*W_d_*) can be, respectively, processed using the following formulas; e.g., $W_{s}=\sqrt{f_{D1}^{2}+f_{D2}^{2}}$, $W_{d}=tan^{-1}\left\{f_{D1}/f_{D2}\right\}$. Here, because the Doppler frequency shift analyzed from the measurement data has a positive or negative sign due to the wind or ocean wave change, the direction component of the wind vector is detectable. In addition, phase unwrapping technique or various digital filter processes can be applied to acquire a stable analysis result. In Figure 20, the analysis accuracy is about RMS error 1.88 m/s for wind speed and RMS error 18.84° for wind direction on average. This analysis result confirms that the wind vector retrieval using the 2-axis Doppler frequency data has enough potential for sea surface monitoring. However, in a point of an accuracy view, further study is needed in detail.

### 6.3. Sea State: (Significant Wave Height and Wind Speed)

**Figure 21:**
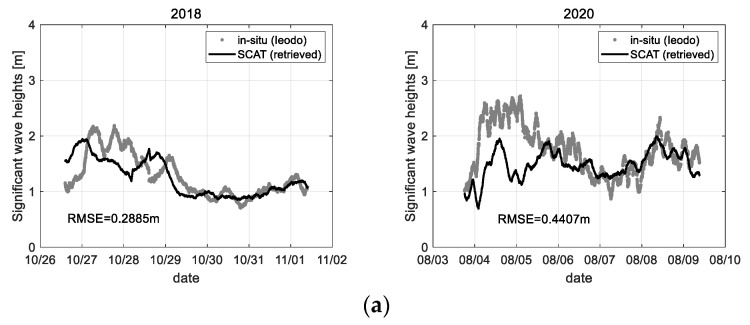

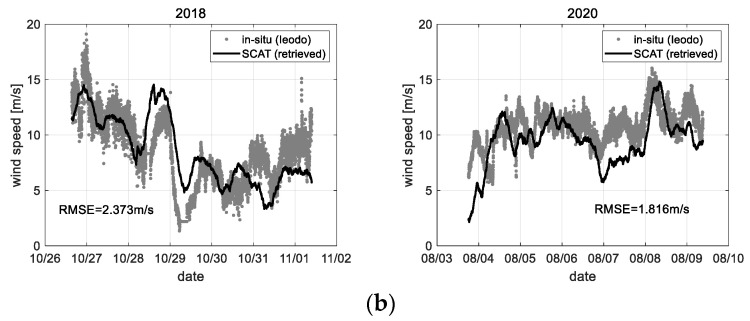
Observational results of (**a**) wind speed and (**b**) significant wave height retrieved by the backscattering coefficient (*σ*°) data of scatterometer system, where 2018, 2020 data only were processed.

Finally, the analysis result of the sea state using the calibrated backscattering coefficient (*σ°*) is shown in Figure 21, where it is based on the correlation to the polarimetric response of microwave from the sea surface (refer to Section 5.3). Figure 21a is the analysis results on the significant wave height, where the analyzed significant wave height includes many errors in a range of more than 1.5 m. Whereas, because the backscattering coefficient of X-band microwave was better correlated to the change of wind speed, Figure 21b shows the analysis results that have a stable signal analysis property overall range of the observed wind speed.

When the calibrated backscattering coefficient data is used for sea state analysis, the retrieval of wind speed has a relatively higher analysis accuracy because the capillary wave is more sensitive to changes in the wind speed and responds better to the X-band microwave. Therefore, we confirmed that the proposed scatterometer system operated at X-band is more sensitive to small scale changes such as the capillary wave, and expect that an observational capability can be enhanced furthermore using this point.

The proposed system and observational data retrieval processes are summarized as shown in Table 4. The distance (*R*) data measured using the altimeter mode can be used for wave height change analysis, and the Doppler frequency (*f_D_*) and backscattering coefficient (*σ°*) measured using the 2-axis scatterometer mode can be also used for sea state or wind vector change analysis. The analysis accuracy of each observation item converted by each radar parameter is about RMSE 0.169 m for tide, 0.127 m for significant wave height, 1.88 m/s for wind speed, and 18.84° for wind direction, respectively. However, in the case of analysis of the significant wave height using the backscattering coefficient, the wind vectors are only detectable in the sea state of a limited range of less than 1.5 m, which corresponds to the WMO sea state code of No 3 or 4.

## 7. Conclusions

The multifunctional scatterometer system for sea surface monitoring and the observation results using the 3 types of radar parameters were verified through the lab- and field tests, which showed enough potential and expandability to observe various oceanographic parameters simultaneously. In a system point of view, it provides not only more functions for sea surface monitoring in comparison to the existing system, but also more measurement data sets applicable for self-calibration and cross-validation. Furthermore, in an ocean observation point of view, it shows the capability for continuous observation of various oceanographic parameters such as tide, significant wave height, and wind speed and direction, which is not required to synchronize for cross-analysis among data sets. In particular, the ocean waves (e.g., tide, significant wave height) converted by distance (*R*) are agreed well with in-situ data because it is directly converted without additional correlation analysis, where it depends on the range resolution only. Whereas the ocean winds converted by Doppler frequency (*f_D_*) or backscattering coefficient (*σ*°) have some constraints to analysis because the inversion of the polarimetric backscattered responses is not intuitive; the scattering model-based inversion algorithm is used in general but, here, we simply compared the correlation between the radar parameter and the oceanographic parameter, then applied for data retrieval. Finally, in a system and data calibration point of view, the existing calibration technique was successfully modified to be optimized for the proposed system with non-standardized data. The precisely calibrated data was verified with the theoretically well-known scattering model, IEM. We confirmed that the analysis result matched well with the backscattering from the relatively smooth sea because the X-band microwave having a short wavelength of about 30 cm reacts better with the capillary wave in the ocean. In summary, the proposed multifunctional scatterometer system has enough observational accuracy in the effective range, however, it is limited to the relatively smooth sea state because of the carrier frequency with a short wavelength. However, it will be overcome by replacing the carrier frequency with the L- or C-band. So, we expect that the proposed system will greatly expand the scope of application of an existing scatterometer system.

## Figures and Tables

**Table 1:** Simulation setup for validating the range-Doppler process.

Parameters	Specification	Notes
Operating frequency (*f*_0_)	9.65 GHz	Bandwidth 500 MHz
Chirp rate (*K_r_*)	500 × 10^9^	(*K_r_* = *BW*/*T_r_*)
Fast-time period (*T_r_*)	1 ms	-
Fast-time (*t*)	1200 samples	(*f_ADC_* = 1.2 MHz)
Slow-time period (*T_η_*)	1 s	-
Slow-time (*η*)	100 arrays	(*f_PRF_* = 100 Hz)
Beat freq.-range ratio (*f_b_*/*R*)	3.33 kHz/m	(*f_r, max_* = *f_ADC_*/2, *R_max_* ≈ 190 m)
Target distance (*R*_0_)	106 m	Incident angle (*θ_i_* = 20°)
Target velocity (*v_t_*)	1 m/s	(*f_D_* = 2*f*_0_·*v_t_*/*c*·sin*θ_i_*)

**Table 2:** Specification of the self-manufactured FMCW scatterometer system.

Radar Parameters	Specifications	Notes
Operating frequency	*f*_0_ = 9.65 GHz, *BW* = 498 MHz	FMCW transceiver
Tx power	~3 Watts	-
Chirp rate (*Kr*)	~498 × 10^9^ Hz/s	(*Kr* ≈ *BW*/*T_r_*)
Fast-time sampling	*T_r_* ≈ 1 ms, *f_ADC_* ≈ 1.2 MHz	(1252 samples)
Slow-time sampling	*T_η_* ≈ 1 s, *f_PRF_* ≈ 100 Hz	(100 arrays)
Incident angle	*θ_i_* = 0°~50°, *ϕ_az_* = 0°, 90°	Motion-control part
Tx/Rx antenna	HPBW (*θ* = 12°, *ϕ* = 10°)	-
Polarization	Vertical	(full-pol. available)

**Table 3:** Example for tidal constituents of the Ieodo ocean area (refer to 2018 data).

Darwin Symbol	M2	S2	N2	K1	O1	M4	M6	MK3	S4	MN4
*H_(n)_*	0.219	0.338	0.385	0.250	0.022	0.007	0.025	0.019	0.009	0.001
*K_(n)_*	175.5°	337.4°	239.3°	211.2°	51.71°	323.6°	105.5°	117.3°	1.11°	14.23°

**Table 4:** Summary of observational data retrieval process (^(1)^ normal-, ^(2)^ oblique-incidence setup).

Radar Parameters	Observation Items	RMSE (‘SCAT’ vs. ‘In-Situ’)	Notes
Distance (*R*)	Tide	0.169 m	^(1)^ Altimeter mode
	Significant wave height	0.127 m	
Doppler frequency (*f_D_*)	Wind speed	1.880 m/s	^(2)^ Scatterometer mode (extended function)
	Wind direction	18.84°	
backscattering coefficient (*σ*°)	Wind speed	2.094 m/s	^(2)^ Scatterometer mode
	Significant wave height	0.365 m	

## Data Availability

Data is contained within the article.

## References

[1] Cavaleri L. (1999). The oceanographic tower Acqua Alta-more than a quarter of century activity. Il Nouovo Cim. C.

[2] Cavaleri L. (2000). The oceanographic tower Acqua Alta-activity and prediction of sea states at Venice. Coast. Eng..

[3] Huang W., Carrasco R., Shen C., Gill E.W., Horstmann J. (2016). Surface current measurements using X-band marine radar with vertical polarization. IEEE Trans. Geosci. Remote Sens..

[4] Carrasco R., Streßer M., Horstmann J. (2017). A simple method for retrieving significant wave height from Dopplerized X-band radar. Ocean. Sci..

[5] Carrasco R., Horstmann J., Seemann J. (2017). Significnat wave height measured by coherent X-band radar. IEEE Trans. Geosci. Remote Sens..

[6] Shim J.S., Chun I.S., Min I.K. Construction of Ieodo ocean research station and its operation. Proceedings of the Fourteenth International Offshore and Polar Engineering Conference.

[7] Choi D.Y., Woo H.J., Park K., Byun D.S., Lee E. (2018). Validation of sea surface wind speed from satellite altimeters and relation to sea state bias–-focus on wind measurements at Ieodo, Marado, Oeyeondo stations. Korean Earth Sci. Soc..

[8] Singh R., Kumar P., Pal P.K. (2012). Assimilation of Oceansat-2-Scatterometer-Derived Surface Winds in the Weather Research and Forecasting Model. IEEE Trans. Geosci. Remote Sens..

[9] Durden S.L., Perkovic-Martin D. (2017). The RapidScat Ocean Winds Scatterometer: A Radar System Engineering Perspective. IEEE Geosci. Remote Sens. Mag..

[10] Malenovský Z., Rott H., Cihlar J., Schaepman M.E., García-Santos G., Fernandes R., Berger M. (2012). Sentinels for science: Potential of Sentinel-1, -2, and -3 missions for scientific observations of ocean, cryosphere, and land. Remote Sens. Environ..

[11] Meta A. (2006). Signal Processing of FMCW Synthetic Aperture Radar Data. Ph.D. Thesis.

[12] Skolnik M. (2002). Indroduction to Radar System.

[13] Skolnik M. (2008). Radar Handbook.

[14] Zaugg E.C., Long D.G. (2015). Generalized frequency scaling and backprojection for LFM-CW SAR processing. IEEE Trans. Geosci. Remote Sens..

[15] Meta A., Hoogeboom P., Ligthart L.P. (2007). Signal Processing for FMCW SAR. IEEE Trans. Geosci. Remote Sens..

[16] Sarabandi K., Ulaby F.T. (1990). A convenient technique for polarimetric calibration of single-antenna radar systems. IEEE Trans. Geosci. Remote Sens..

[17] Sarabandi K., Ulaby F.T. (1992). Measurement and calibration of differential mueller matrix of distributed targets. IEEE Trans. Anrennas Propag..

[18] Fung A.K., Li Z., Chen K.S. (1992). Backscattering from a randomly rough dielectric surface. IEEE Trans. Geosci. Remote Sens..

[19] Fung A.K., Chen K.S., Chen K.S. (2010). Microwave Scattering and Emission Models for Users.

[20] Korea Hydrographic and Oceanographic Agency, Ieodo Ocean Research Station http://khoa.go.kr/ors/station_intro1.do.

[21] Carrara W.G., Goodman R.S., Majewski R.M. (1995). Spotlight Synthetic Aperture Radar: Signal Processing Algorithm.

[22] Hwang J.H., Lee K.Y., Park S.M., Oh Y.S. (2009). Design of a full polarimetric scatterometer for X-band. J. Korean Inst. Electromagn. Eng. Sci..

[23] Hwang J.H., Park S.M., Kwon S.G., Oh Y.S. (2010). Study on the calibration of a full-polarimetric scatterometer system at X-band. J. Korean Inst. Electromagn. Eng. Sci..

[24] WMO Sea State Code. http://en.wikipedia.org/wiki/Seastate#WMOseastatecode.

[25] Jin Y.Q. (2006). Theory and Approach of Information Retrievals from Electromagnetic Scattering and Remote Sensing.

[26] Kanevsky M.B. (2008). Radar Imaging of the Ocean Waves.

